# Urinary Tract Infection in Children With Bronchiolitis: Is It Worth Testing Everyone?

**DOI:** 10.7759/cureus.15485

**Published:** 2021-06-06

**Authors:** Tasnim R Edun, Omar Aldibasi, Syed F Jamil

**Affiliations:** 1 Pediatrics, King Abdullah Specialized Children's Hospital, King Abdulaziz Medical City, Ministry of National Guard Health Affairs, Riyadh, SAU; 2 Biostatistics, King Abdullah International Medical Research Center, Riyadh, SAU; 3 Pediatrics, College of Medicine, King Saud bin Abdulaziz University for Health Sciences, Riyadh, SAU

**Keywords:** uti, bronchiolitis, urinalysis, urine culture, urinary tract infection

## Abstract

Objective

The current study aims to determine the prevalence of urinary tract infection (UTI) and the need to perform urine analysis and cultures in children admitted with bronchiolitis in a large tertiary children’s hospital in Riyadh, Saudi Arabia.

Methods

We conducted a retrospective chart review of pediatric patients 0-2 years of age who were admitted with bronchiolitis from November 2016 till April 2017. All charts were analyzed to identify the children investigated for UTI, and their results were then reviewed.

Results

There were 407 children admitted with bronchiolitis during the study period. Two-thirds of them were investigated for UTI. Only 2.6% of the patients tested positive for urine culture, and only 0.96% were found to have a true UTI.

Conclusion

The prevalence of UTI in children with bronchiolitis is too low to justify routine screening. Therefore, only children with a high risk of having UTI should be investigated.

## Introduction

Bronchiolitis is a common cause of lower respiratory tract infection in children below two years of age and contributes to a significant proportion of hospital stay in peak season.

According to the American Academy of Pediatrics (AAP), bronchiolitis is defined as a cluster of clinical symptoms and signs that include “a viral upper respiratory prodrome followed by increased respiratory effort and wheezing in children less than two years of age” [1].

There is a wide range of prevalences reported in previous research studies looking at the risk of concurrent urinary tract infection (UTI) in patients with bronchiolitis, with rates ranging from 0 [2,3] to 12% [4]. Physicians tend to err on the side of caution due to the complications associated with UTI and, therefore, often routinely test for UTI in children who present with bronchiolitis.

To diagnose a UTI, the AAP Subcommittee on Urinary Tract Infection recommends that the following two parameters should be fulfilled: (1) a urinalysis results suggesting infection (positive leukocyte esterase or nitrite or microscopic analysis showing positive results for leukocytes or bacteria) and (2) a culture of a urine specimen obtained via suprapubic aspiration or urinary catheterization showing the presence of ≥50,000 colony-forming units (CFUs) per millilitre of a uropathogen [5].

While the AAP recommendation is for children aged 2 to 24 months, urinalysis has also been found to be highly sensitive and specific for diagnosing UTIs in febrile infants 60 days old or younger [6].

The AAP also recommends that UTI testing is warranted when the risk for UTI is between 1% and 3% in children presenting with bronchiolitis [5].

We noticed that that urine investigations via urinary catheterization are routinely done for patients with bronchiolitis in our Pediatric Emergency Department. Therefore, we decided to determine the prevalence of UTI in children admitted for bronchiolitis in our tertiary care children’s hospital. We aimed to establish whether urine investigations should continue to be carried out routinely in children diagnosed with bronchiolitis.

## Materials and methods

We conducted a retrospective cross-sectional study of pediatric patients 0-2 years of age who were hospitalized with a diagnosis of bronchiolitis in the Department of Pediatrics, King Abdullah Specialized Children's Hospital, Ministry of National Guard Health Affairs, Riyadh, Saudi Arabia. The definition of bronchiolitis that is used in this study is the presence of respiratory signs and symptoms such as rhinorrhea, cough, tachypnea and wheezing, associated with increased respiratory efforts such as nasal flaring, grunting, subcostal and/or intercostal retractions in children less than two years of age [1]. The study period was November 2016 to April 2017, which tends to be the peak season of upper respiratory infections. We excluded the children who were older than two years at the time of admission and if admitted to other departments such as Pediatric Cardiology. A search was done in Best Care, our electronic health information system. The data collected included age at admission, urine investigations performed, and urine culture results. We used both a positive urinalysis and a positive culture to diagnose UTI as per the criteria recommended in AAP Clinical Practice Guidelines [5]. If the urine culture was positive, i.e., showing the presence of ≥50,000 CFUs per millilitre of a uropathogen, we also looked into the urinalysis of those patients. We considered a positive urinalysis to be at least one of the following: with white blood cells ≥5 /high-power field, nitrite positive, leucocyte esterase positive or with the presence of bacteria.

Statistical analysis

Descriptive statistics were applied to characterize the study participants' variables in terms of frequencies, percentages or means, and standard deviations. In the univariate analysis, we compared the study participants' variables using the chi-square tests at a level of significance (α=0.05) for the urine investigations. Statistical analysis was conducted using SAS 9.4 (SAS Institute Inc., Cary, NC, USA) and data visualization using MS Excel.

Ethical consideration

Our study was approved by the Institutional Review Board (IRB) at King Abdullah International Medical Research Center (Ref.RC16/141/R).

## Results

**Figure 1:**
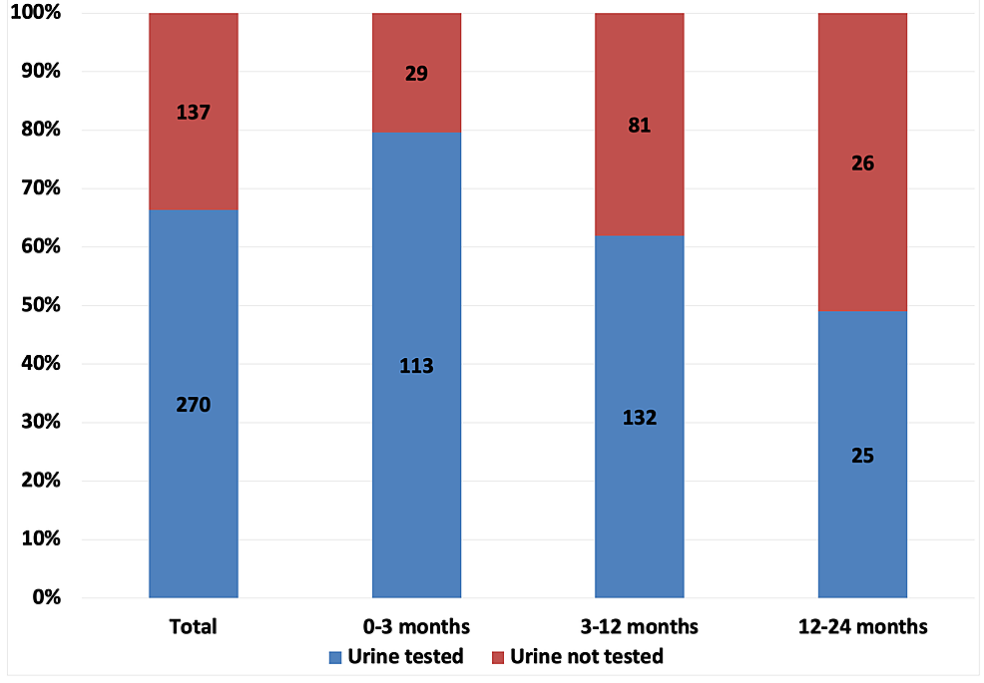
Urine tests according to age groups

**Figure 2:**
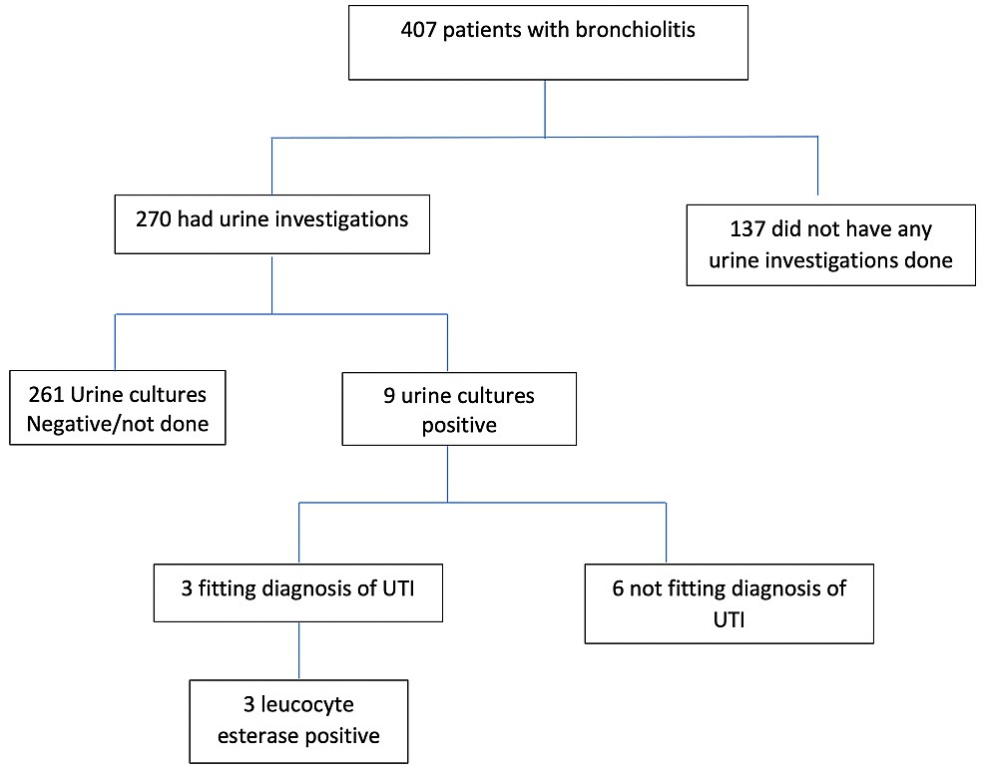
Patients with bronchiolitis and UTI UTI, urinary tract infection

A total of 407 children fulfilled the inclusion criteria. The mean age at admission was 5.69 ± 4.94 months. There was a slight preponderance of males 246 (59.46%) compared to females 165 (40.54%). Urine investigations were done in 270 (66.34%) of the patients. These were carried out almost equally in males (64.88%) and females (68.48%) (chi-square: p=0.45). However, they varied across age groups (Figure 1), with the 0- to 3-month age group more likely to be tested (79.58%, chi-square: p<0.0001). Testing was done in 61.97% (chi-square: p<0.0001) of the 3- to 12-month group and in 48.08% (chi-square: p<0.0001) in the 12- to 24-month age group. Nine (2.2%) had positive urine cultures, out of which only 3 (0.74%, confidence interval 0.15%, 0.214%) had positive urinalysis. None of the urinalysis was nitrite positive or showed the presence of bacteria. They all were leucocyte esterase positive (Figure 2).

## Discussion

To our knowledge, this is the first study in Saudi Arabia to investigate the prevalence of concurrent UTI in bronchiolitis. We found that there was a tendency to test younger children more than required. Out of 270 children tested for urine, only 1.1% had a UTI. This gives the prevalence of a UTI to be less than 1% based on testing two-thirds of all diagnosed bronchiolitis cases.

Our study concurs with many previous studies that the rate of concurrent infections is not significant. Some studies mentioned that there were no patients with UTI in bronchiolitis or in a subset of respiratory syncytial virus (RSV) bronchiolitis patients admitted to ICU [2,3,7].

Ahmad et al. conducted a study of 141 infants aged less than 90 days diagnosed with bronchiolitis and reported culture-positive prevalence of UTI as 0.71% [8]. However, the study did not mention whether urinalysis was done. More recently, Schlecther Salinas et al. [9] reported a UTI rate of 0.49% in febrile infants aged 2-12 months with influenza or RSV infection, while other studies have shown a UTI rate of about 2% [10,11].

In contrast, many studies are revealing a high prevalence rate, ranging from 4% to 8% [12-17]. Some even reported a rate of UTI of 10% or more [4,18].

One of the reasons for the wide range of concurrent UTI reporting in bronchiolitis is the lack of consensus on UTI diagnostic criteria. Many of the earlier studies did not have a consistent definition for the diagnosis of UTI. The definition of a positive urine culture was not mentioned in some studies and was different across published studies [17]. For example, several studies used a positive culture of more than 10,000 CFUs of a single pathogen as their diagnostic criteria for UTI [10,11,16], while Kaluarachchi had three different criteria for UTI diagnosis: “the growth of a single known pathogen with colony counts meeting 1 of 3 criteria: (1) 1000 colony-forming units (CFU)/mL or greater for urine cultures obtained by suprapubic aspiration, (2) 50,000 CFUs/mL or greater from a catheterized specimen, or (3) 10,000 CFUs/mL or greater from a catheterized specimen in association with a positive urinalysis finding” [15].

Many studies did not use urine analysis (UA) as a criterion for diagnosing UTI [2,4,7,10-12,17]. Melendez and Harper reported the prevalence of UTI to be 2.2% while using the diagnostic criteria of a positive culture of more than 10,000 CFUs of a single pathogen [11]. This would be equivalent to 1.8% if using the diagnostic criteria of 50,000 CFUs, and only 0.4% when using the AAP guidelines criteria.

A systemic review by Ralston et al. on occult serious bacterial infection in infants younger than 60 to 90 days diagnosed with bronchiolitis concluded that the average rate of UTI was 3.3% (95% CI 1.9%-5.7%) but admitted that the rate could have been confounded by asymptomatic bacteriuria [19]. Moreover, McDaniel et al., in their systematic review and meta-analysis, showed that out of 18 studies reporting the prevalence of UTI in bronchiolitis, 11 did not include UA as part of their criteria to diagnose UTI [20]. If considering all the 18 studies, the prevalence of UTI in bronchiolitis was 3.1% (95% CI 1.8-4.6). However, when the diagnosis of UTI required a positive UA, the prevalence of UTI was 0.8% (95% CI 0.33-1.40), which is less than AAP guidelines thresholds for testing.

Another reason for the difference in reported concurrent UTI rates is the inclusion criteria used. Different age groups were used in different studies. Some investigated only patients with RSV bronchiolitis or only patients with a certain degree of temperature. While some studies include all patients with bronchiolitis, others include only patients who were tested. Including only tested patients can be a bias as some physicians might tend to test those suspected to have sepsis or a risk factor, and different physicians might have different thresholds for testing.

Yet another explanation for the discrepancy is that the children tested might have a high probability for UTI based on their own individual risk factors, as mentioned in the AAP guidelines. For instance, Elkhunovich et al. reported that all the males who tested positive for UTI were not circumcised [14]. Moreover, Schlechter Salinas et al. found out that all patients who tested positive for UTI had a risk factor of >1% [9].

Strengths and limitations

There are several strengths of our study. To our knowledge, this is the first study in Saudi Arabia focusing on investigating the prevalence of concurrent UTI in bronchiolitis. Moreover, we used the AAP definition for UTI (positive urinalysis and urine culture) to reduce false positivity.

Although our findings are based on a single tertiary center experience, results are likely to be generalizable as consistent with previous studies.

Our study limitations are that, as it was a retrospective study, we do not know whether there were specific symptomatic criteria that led to urine investigations in some patients and also if the males were circumcised or not.

## Conclusions

Physicians tend to routinely test for UTI in patients with bronchiolitis. Our study shows that 66% of the patients with bronchiolitis were tested for UTI but less than 1% had confirmed UTI. This is below the recommended threshold for testing by AAP. Routine testing causes discomfort and pain to the patients and unnecessary stress to parents, at the same time increasing health care costs, without any benefit.

Hence, when contemplating whether to test a patient for UTI, individual risk factors should be considered. Further education of physicians and reinforcement are needed to change the current practice.
